# Research on Classification of Open-Pit Mineral Exploiting Information Based on OOB RFE Feature Optimization

**DOI:** 10.3390/s22051948

**Published:** 2022-03-02

**Authors:** Yi Zhou, Shufang Tian, Jianping Chen, Yao Liu, Chaozhu Li

**Affiliations:** 1 School of Earth Sciences and Resources, China University of Geosciences (Beijing), Beijing 100083, China; zhouyi18@cugb.edu.cn (Y.Z.); 3s@cugb.edu.cn (J.C.); 2 Command Center of Natural Resources Comprehensive Survey, China Geological Survey, Beijing 100055, China; lichaozhu@cags.ac.cn; 3 Land Satellite Remote Sensing Application Center, Ministry of Natural Resources of China, Beijing 100048, China; liuyao@lasac.cn

**Keywords:** feature optimization, mineral exploiting information, remote sensing, image classification, machine learning

## Abstract

Mineral exploiting information is an important indicator to reflect regional mineral activities. Accurate extraction of this information is essential to mineral management and environmental protection. In recent years, there are an increasingly large number of pieces of research on land surface information classification by conducting multi-source remote sensing data. However, in order to achieve the best classification result, how to select the optimal feature combination is the key issue. This study creatively combines Out of Bag data with Recursive Feature Elimination (OOB RFE) to optimize the feature combination of the mineral exploiting information of non-metallic building materials in Fujian province, China. We acquired and integrated Ziyuan-1-02D (ZY-1-02D) hyperspectral imagery, landsat-8 multispectral imagery, and Sentinel-1 Synthetic Aperture Radar (SAR) imagery to gain spectrum, heat, polarization, and texture features; also, two machine learning methods were adopted to classify the mineral exploiting information in our study area. After assessment and comparison on accuracy, it proves that the classification generated from our new OOB RFE method, which combine with random forest (RF), can achieve the highest overall accuracy 93.64% (with a kappa coefficient of 0.926). Comparing with Recursive Feature Elimination (RFE) alone, OOB REF can precisely filter the feature combination and lead to optimal result. Under the same feature scheme, RF is effective on classifying the mineral exploiting information of the research field. The feature optimization method and optimal feature combination proposed in our study can provide technical support and theoretical reference for extraction and classification of mineral exploiting information applied in other regions.

## 1. Introduction

The improper exploitation and utilization of mineral resources may mainly be responsible for the deterioration of ecological environments worldwide. Mineral exploiting information, which generally refers to the scope of the mining area and its status (using/discarded/restored), is a basic indicator to monitor mineral activities and evaluate ecological conditions in a regional mining area [1,2,3]. Therefore, collecting mineral exploiting information timely and accurately can provide a solid database for effective mining activity management and ecological restoration [4]. Comparing with traditional field research, remote sensing has several advantages, like high timeliness, extensive coverage, and immediate results; and because of all the above has been applied in mineral exploitation since 1970s [5,6]. Optical image is the most commonly used data source to obtain mineral exploiting information. For instance, the China Geological Survey adopted high-resolution remote sensing images to monitor the status of mining areas and land use from 2003 [7]. Also, Felipe de Lucia Lobo et al. graphed the landscape of a mining area in Amazon, Brazil, based on multispectral data obtained from supervised classification with a kappa coefficient of 0.7 [8]. Using Landsat multispectral data, Lifeng Xie et al. extracted multiple ecological index and established mining and restoration assessment indicators (MRAIs), which are applicable in data collection and supervision of restoration in mining areas of southern JiangXi province, China [9]. Li Hengkai et al. applied Sentinel-2 images to research the devastation and restoration of rare earths regions [10]. Due to the similarity of the spectral characteristics between mines under different developing status and other land features, it is likely to affect the accuracy of land-feature classification and difficult to gather further useful information, if we relied solely on single spectral image [11,12,13]. Moreover, manual calibration is always required to obtain more precise results [14,15,16]. There are studies using Polarimetric Synthetic Aperture Radar (PolSAR) data to classify mineral ground features successfully, which may prove the feasibility of Synthetic Aperture Radar (SAR) applied to mine classification as well [17]. In addition, for mines and other land features related with human activities like residential buildings and transportation, land surface temperature (LST) is the key factor to distinguish them [18] and can be captured by thermal infrared data. However, thermal data is seldom used in mine classification because of its low spatial resolution [19].

Differing from using a single data source, combining different data of land features can fully amplify the respective advantages of those combinations, so it is more likely to achieve practical success in research. Specifically, much researchers has already combined hyperspectral, multispectral, SAR data, and the like together to identify vegetation and evaluate wetlands and soil quality; and made great progress in these research pieces. Additionally, the overall accuracy of the classification has been significantly improved [20,21,22]. The current focus of multi-source imagery classification is to optimally select feature combination [23], as too many features probably weaken the performance of the classifier and lead to unfavorable low accuracy and inefficiency [24]. Meanwhile, for classification of mineral exploiting information, it is quite challenging to determine the most optimal and relevant parameter, with logic and efficiency, from numerous available features. In the past, researchers test all possible feature combinations and then determine the most suitable ones, but it is obviously time-consuming for researchers with forced-repetitious works. To deal with this problem, at present, Recursive Feature Elimination (RFE) is feasible to optimize feature combinations by intentionally omitting particular features through recursion, then building model on rest data and ultimately filtering the optimal combination based on the previous modeling results. As a result of all advantages and conveniences attributed to RFE, it is now widely adopted in land use classification, biological information identification, landslide sensitivity assessment, etc. [25,26,27]. To explain, RFE can intentionally omit particular features through recursion, build model on rest data, and filter the optimum combination based on modeling results. Nevertheless, in practical application of RFE, there is a potential data leakage problem in RFE when selecting the preferred number of optimal features [28], which may lead to greater variance on results and over-fitting on the training model [29]. Therefore, to prevent the leakage problem from disturbing our research accuracy, we created Out of Bag data with Recursive Feature Elimination (OOB RFE) feature combination optimization method that tested and verified each feature combination by applying OOB samples in RFE. We expected the new method could remedy the shortage in RFE and reinforce the generalization ability of the model to increase the accuracy further. 

The study set the non-metallic building material ore-concentrated area in southern Fujian, China as research field, using Ziyuan-1-02D (ZY-1-02D) hyperspectral, Landsat-8 multispectral and Sentinel-1 SAR data to extract 52 features in total, like spectrum, heat, polarization, texture, etc. We also applied support vector machine (SVM), random forest (RF) on different feature combinations to classify mining information, and finally conducted accuracy verification and comparison. The study aimed to testify the validity of OOB RFE method in research of mineral exploitation, and to provide a new idea for the selection and optimization of feature variables in mineral exploiting information classification.

## 2. Materials and Methods

### 2.1. Study Area

**Figure 1:**
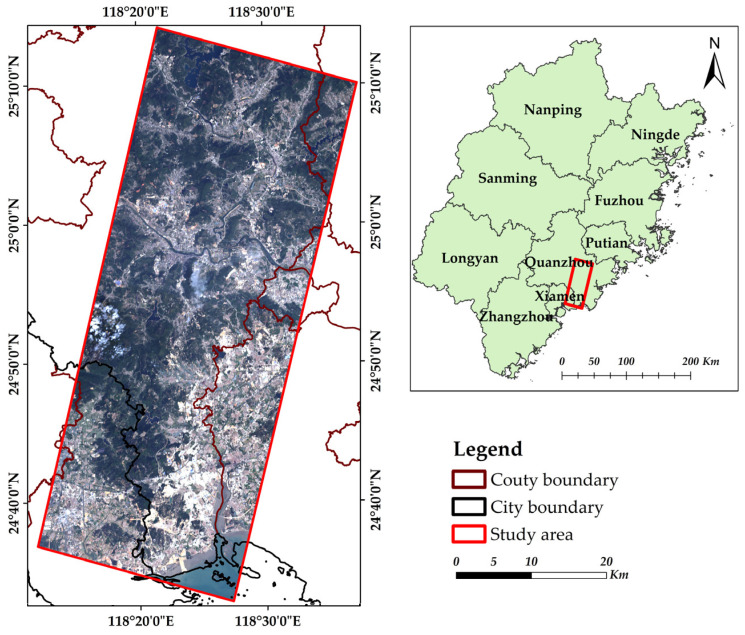
Location of the study area and map shown in true color image from Ziyuan-1-02D (ZY-1-02D).

The study area is located in the southern part of Quanzhou City, Fujian Province, China (Figure 1). This area is rich in non-metallic minerals for building materials, and thus one of the most important mineral areas in China. There is high-frequency of open-pit mining in this particular area to mainly exploit limestone for construction. It is valuable to conduct classification of mineral exploiting information in the typical area to facilitate the mining process because of various mining area under development and relative lack of effective exploiting methods.

### 2.2. Data and Preprocessing

#### 2.2.1. Satellite Images

The 2 scenes of ZY-1-02D hyperspectral image data used in this study were taken on 16 March 2020. In order to ensure the data accuracy and the consistency of land features and corresponding timeline, 1 Landsat-8 image (https://earthexplorer.usgs.gov/, accessed on 26 October 2021) and 1 Sentinel-1 image (https://scihub.copernicus.eu/, accessed on 26 October 2021) were taken on 16 March 2020 and 17 March 2020, respectively. Specific information is shown in Table 1.

#### 2.2.2. Auxiliary Data

**Figure 2:**
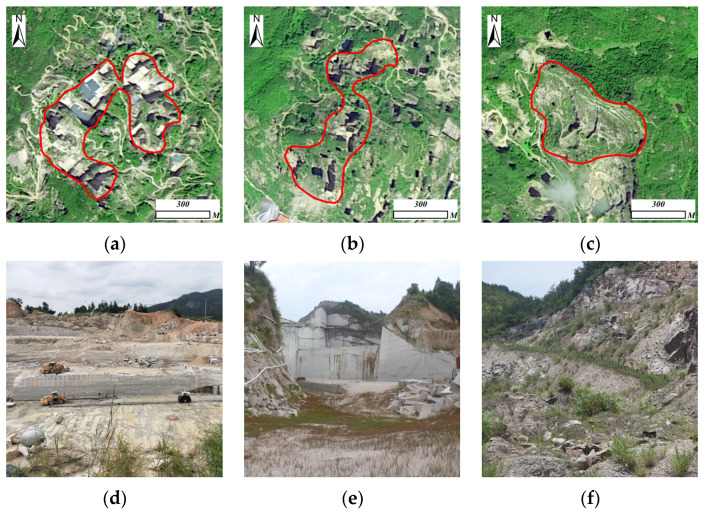
Satellite imagery (from GaoFen-2 dataset) and corresponding field photo of 3 typical exploiting states’ mines in the study area: (**a**,**d**): Using mine; (**b**,**e**): Discarded mine; (**c**,**f**): Restored mine.

The auxiliary data includes: Land Use and Land Cover (LULC) data and measured data from fieldwork. LULC data was generated from experts’ visual interpretation based on GaoFen-2 high-resolution datasets (photographing time range stretches from November 2019 to May 2020). To ensure the data accuracy, this study collected field survey data separately in December 2019 and June 2020, including mineral exploiting status and land-featured information in the study area (Figure 2); all collected data were updated and correlated with each other in LULC data.

#### 2.2.3. Classification Types

Integrating the previous research [30] with our purpose, the land cover feature in this study were classified into mines, building lots, croplands, forests, bare lands, and waterbodies; furthermore, it is noteworthy that we divided the mines’ status to using, discarded, and restored.

#### 2.2.4. Data Preprocessing

First, radiometric calibration and atmospheric correction were implemented on Landsat-8 and ZY-1-02D data; meanwhile we used Shuttle Radar Topography Mission (SRTM) 30-m Digital Elevation Model (DEM) data as supplement and adopted C-correction model (Formula (1)) to achieve terrain correction on ZY-1-02D, in order to reduce the effect of terrain complexity on land-featured spectral information.

(1) $$L_{H}=L_{T}\frac{\cos\left(\theta \right)+c}{\cos\left(i\right)-c}$$

where $L_{H}$ is the corrected image value, $L_{T}$ is the uncorrected image value, θ is the solar zenith angle, i is the solar incident angle concerning the surface normal direction, and c is the C-correction coefficient derived by regressing $L_{T}$ against cos(i) and taking the quotient of the intercept and slope.

Then, there are six preprocessing toward Sentinel-1A data through SNAP: (1) Orbit correction; (2) Thermal noise removal; (3) Radiation calibration; (4) Coherent speckle filtering; (5) Terrain correction; (6) Decibelization. 

We also use bilinear interpolation to resample the processed Sentinel-1 data to 30 m resolution, which is consistent with the resolution of ZY-1-02D and Landsat-8 data.

Finally, 47 ground control points were selected for image registration of the above three types of images, so the spatial error can be controlled within 0.5 pixels.

## 3. Methods

**Figure 3:**
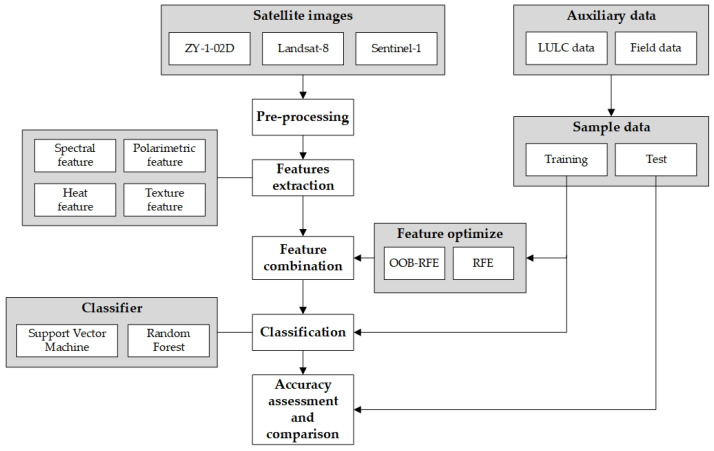
Processing workflow of this study.

The workflow is shown in Figure 3. First, after preprocessing three types of remote sensing data, we extracted spectral, thermal, polarization, and texture features, and established different combinations through OOB RFE and RFE feature optimization. Then, SVM, RF were applied to classify, and finally we conducted accuracy verification and comparison.

### 3.1. Feature Extraction

Filtering appropriate features based on the characteristics of data can make sure that the data is fully utilized, meanwhile, which can ensure efficient classification and accuracy [31]. This study extracts the following features according to 3 types of data [32,33,34].

#### 3.1.1. Spectral Feature

In order to acquire feature consistency, all spectral features in this study are extracted by ZY-1-02D. Due to the Hughes effect, hyperspectral images are not directly used in land cover classification [35]. Improving on previous studies [20,21], we selected 3 kinds of features including Minimum Noise Fraction (MNF), band, and index features. 

##### Minimum Noise Fraction

Minimum Noise Fraction (MNF) plays a pivotal role in dimensional reduction and reconstruction for hyperspectral data. It can display important features on band images collectively, which means it can effectively eliminate the data noise and reduce the bands’ correlations. By doing so, inefficient calculations can be avoided in the classification and indistinguishable features will be revealed [36]. In this study, MNF was performed on ZY-1-02D, and the first 2 components of MNF were selected as the MNF features.

##### Band and Index Feature

**Figure 4:**
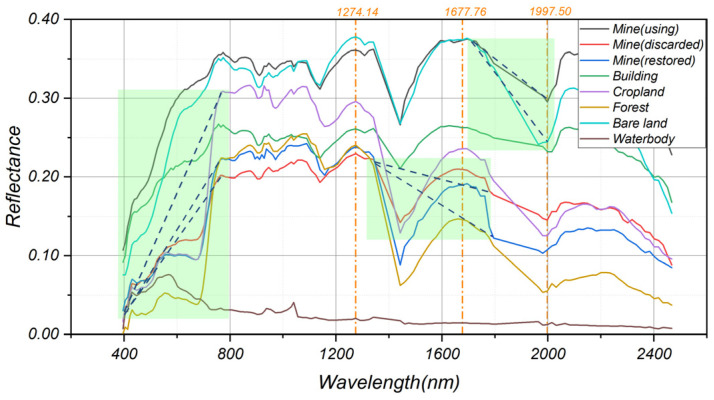
Spectral characteristic curve of 8 land cover types from ZY-1-02D in the study area.

Through spectral curve analysis (Figure 4), we found that mines under different status has similarities with other landscapes regarding spectral features, whereas there is high heterogeneity of spectral reflectance values among different landscapes on Band 93 (1274.14 nm), Band 112 (1677.76 nm), and Band 122 (1997.50 nm) (vertical orange dotted-line on Figure 4). Thus, these 3 particular bands were chosen in the classification to distinguish the mines from other landscapes, meanwhile, we created 3 mine indexes (MI) by extracting slopes of different bands (light green area on Figure 4):

(2) $$MI1=\left(\rho \left(765.44\;\mathrm{nm}\right)-\rho \left(395.86\;\mathrm{nm}\right)\right)/\Delta \lambda_{1}$$

(3) $$MI2=\left(\rho \left(1341.15\;\mathrm{nm}\right)-\rho \left(1762.16\;\mathrm{nm}\right)\right)/\Delta \lambda_{2}$$

(4) $$MI3=\left(\rho \left(1711.52\;\mathrm{nm}\right)-\rho \left(1997.50\;\mathrm{nm}\right)\right)/\Delta \lambda_{3}$$

where, ρ is the spectral reflectance value of the corresponding band; $\Delta \lambda_{1}$, $\Delta \lambda_{2}$, and $\Delta \lambda_{3}$ are respectively the wavelength difference of 395.86–765.44 nm, 1341.15–1762.16 nm, and 1711.52–1997.50 nm.

In addition, referring to the Landsat-8 Operational Land Imager (OLI) band characteristics, 6 main band values are extracted from ZY-1-02D (Table 2). Since ZY-1-02D hyperspectral data has a relatively low signal-to-noise ratio, calculation using a single band is susceptible to noise [37], so in this study, we calculated the average surface reflectance by combining different bands and set the average value as a band variable.

Moreover, 3 kinds of spectral indices [38,39] commonly used for feature classification are calculated by the same average value (Table 3).

**Figure 5:**
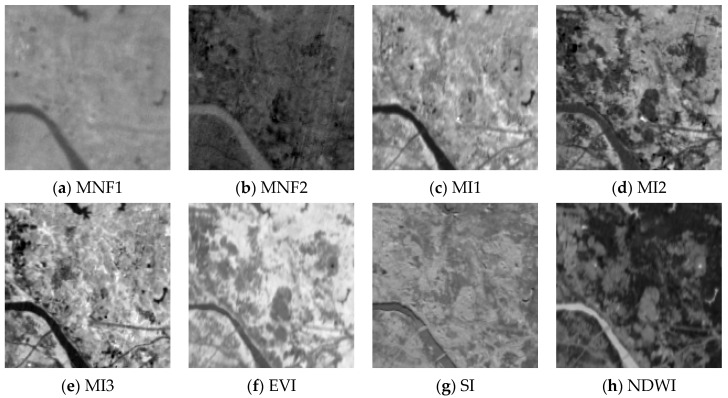
The spectral index feature map of local study area. MNF1: Minimum Noise Fraction feature 1; MNF2: Minimum Noise Fraction feature 2; MI1: mine index 1; MI2: mine index 2; MI3: mine index 3; EVI: Enhanced Vegetation Index; SI: Soil Index; NDWI: Normalized Difference Water Index.

Figure 5 shows the local spectral index feature map of the study area.

#### 3.1.2. Heat Feature

Landsat-8 has Thermal Infrared Sensor (TIRS) data recorded the thermal infrared information of the ground features. The surface temperature data obtained from the data inversion basing on TIRS are already successfully applied to artificial feature recognition and surface thermal status evaluation [40,41]. The atmospheric correction method (also called Radiative Transfer Equation (RTE)), which is widely adopted to extract LST through Landsat-8 data attributing to its high accuracy and feasibility, is used in our study to extract surface heat characteristics [42].

The calculation of RTE is shown below:

(5) $$L_{\lambda }=\left[\varepsilon B\left(T_{S}\right)+\left(1-\varepsilon \right)L↓\right]\tau +L↑$$

where, $L_{\lambda }$ is the thermal infrared radiance value received by the satellite sensor, ε is the surface specific emissivity, $T_{S}$ is the true surface temperature (K), $B\left(T_{S}\right)$ is the black body thermal radiance, and τ is the transmittance of the atmosphere in the thermal infrared band.

*B*(*T_S_*) can be calculated by RTE, and *T_S_* can be obtained by the Planck formula function:

(6) $$T_{S}=K_{2}/\ln(K_{1}/B\left(T_{S}\right)+1)$$

for Landsat-8 band10, $K_{1}$ = 774.89 W/(m^2^ × µm × sr), $K_{2}$ = 1321.08 K.

**Figure 6:**
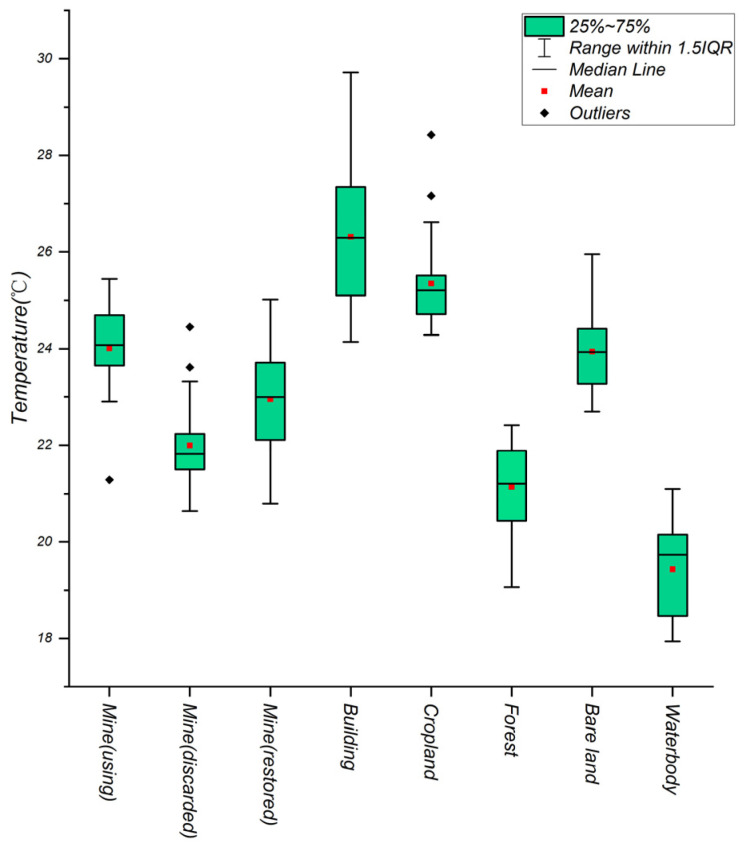
Land surface temperature statistics of different land type.

The surface temperature statistics of different features based on sample data are shown in Figure 6.

#### 3.1.3. Polarimetric Feature

**Figure 7:**
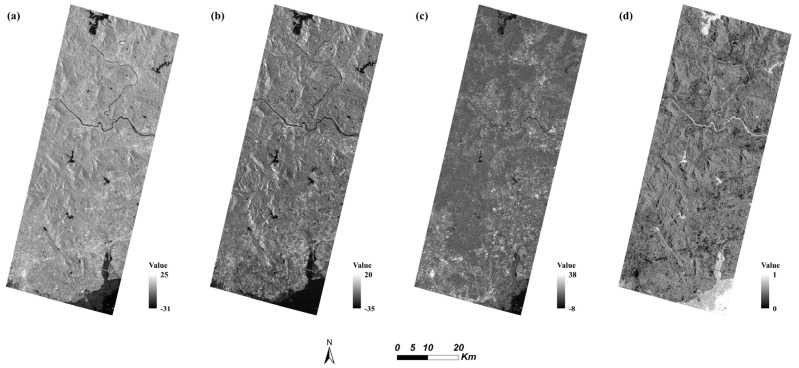
Polarimetric feature map of the study area from Sentinel-1 image: (**a**) VV band, (**b**) VH band, (**c**) VV-VH, (**d**) VV/VH.

SAR data can reflect the geometric and dielectric characteristics scattered by ground objects and enable us to improve the precision of classification [43]. This study acquired 4 polarization features from Sentinel-1 data (Figure 7).

#### 3.1.4. Texture Feature

Important imagery information can be obtained from texture features [44]. Gray-level co-occurrence matrix (GLCM) is a method that utilize relative angles of gray-scale space between pixels to extract texture information, and thus is widely used in the land cover information extraction based on satellite imagery [45]. Applying with GLCM, this study collected a total of 30 texture variables, like contrast, dissimilarity, homogeneity, Angular Second Moment (ASM), variance, etc., by calculating two ZY-1-02D MNF images and four sentinel-1 polarization images.

### 3.2. Feature Optimization

The main purpose of feature optimization is to remove redundant and irrelevant information before classification and prediction, reinforce generalized ability of the model, and to increase classification accuracy at the same time.

#### 3.2.1. Recursive Feature Elimination

Recursive Feature Elimination (RFE) is a greedy algorithm that can help researchers find the optimal feature subset that can maximize the model function through eliminating certain feature variables. To be specific, the principle of RFE is explained below: whole data set is enter into RFE as start point, and then, based on the prediction accuracy of the classifier (base model, like SVM, Logistic, etc.), RFE can use accuracy gained from the base model to evaluate the relevance of each feature and remove the feature with lowest relevance in each loop iteration until the remaining data set is empty. Also, the elimination sequence of each feature demonstrates its importance toward combination, so we can easily sort those features by their importance [46]. RFE requires cross-validation to select the optimal number of features: Given that a set includes d features, then all subsets it included can be written as 2^d^ − 1 (including the empty set). After calculating the validation error of each subset using basic model, this method chooses subset with the smallest error as the required feature [47].

**Figure 8:**
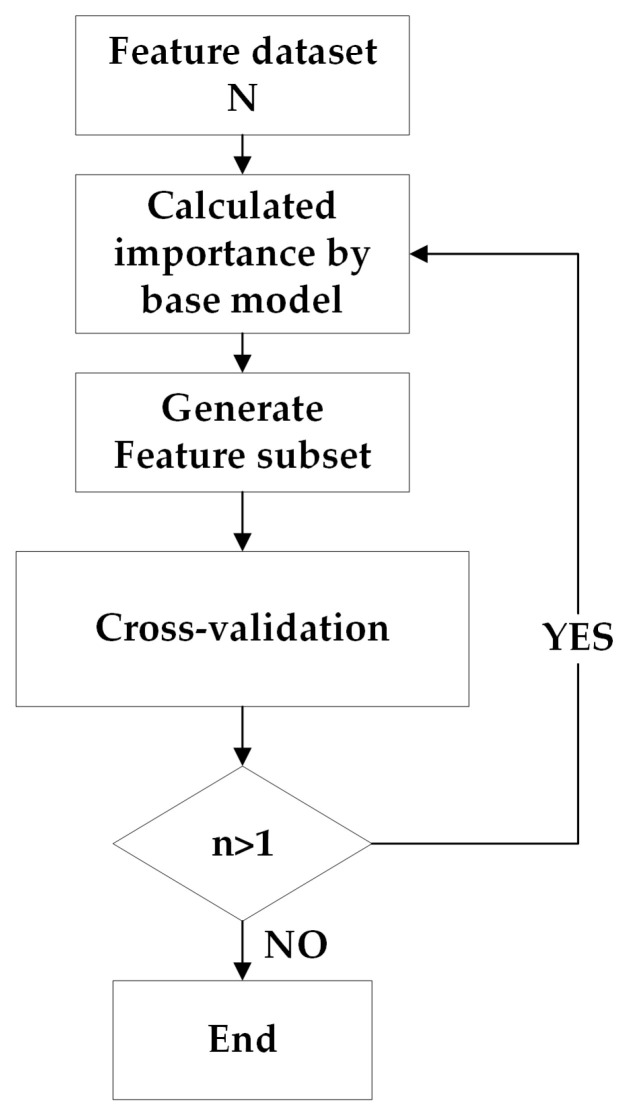
Flow chart of Recursive Feature Elimination (RFE).

The common work flow of RFE is shown below (Figure 8):

(1) Pick an initial feature dataset N with n features and choose a base model for RFE;
(2) Generate feature subset by removing the features with lowest score based on calculation of base model;
(3) Basing on base model, deviation of subset can be testified through cross validation;
(4) Repeat step (2), (3), until the last feature was left over. After comparing every output, the feature subset with smallest deviation can be considered as the optimal feature set.

Overall, RFE has 2 big advantages: eliminating irrelevant features while ensuring accuracy and avoiding the degradation of classification performance caused by data redundancy [48]. However, it is noteworthy that there is a potential data leakage problem through cross-validation based on RFE: as every feature from the whole dataset was involved in the validation, which means those features might be seen by some decision tree or even applied in training models, there can be great variance in validation and over-fitting in results generated by this method. By over-fitting it means that result of training model shows high accuracy, whereas the weak results may occur in practice [49,50]. To tackle the problem, we have to find a more appropriate method to avoid the potential data leakage problem brought by cross validation in traditional RFE and to determine the optimal feature combination.

#### 3.2.2. OOB RFE

Out of Bag data (OOB) means that when we use bootstrap sampling (random sampling with put-back) in random forest to generate the training set of decision tree, some samples (about 1/3) are never put into the sampling set of the decision tree. We call the unselected samples as OOB [51]. OOB error and OOB score obtained from OOB can be, respectively, used to evaluate feature importance and feature dimensional performance.

The principle of using OOB error to evaluate the importance of features is explained below. Among a forest composed of *n* decision trees, first, we based it on OOB data to calculate the Out of Bag error of each decision tree in the random forest, and then randomly changed the *j*-th feature variable of the Out of Bag data to get the new Out of Bag error; The more the Out of Bag error increases and accuracy rate decreases, the more important the variable is. The importance of is expressed as [52]:

(7) $$V\left(X^{j}\right)=\frac{1}{n}\sum_{t=1}^{n}\left(e_{t}^{j}-e_{t}\right)$$

OOB score refers to the Out of Bag estimation accuracy score, namely, the proportion of correct predictions for Out of Bag samples. Since OOB data was not involved in the construction of the decision tree, it can be used to evaluate the results of the regression model. Usually, we use mean square error predicted by OOB to evaluate the effect of model estimation [53]:

(8) $$MSE_{OOB}=\frac{1}{n}\overset{n}{\underset{1}{\sum }}{\left(y_{i}-y_{i}^{OOB}\right)}^{2}$$

where, $y_{i}$ is the actual value of the dependent variable in the Out of Bag data, and $y_{i}^{OOB}$ is the predicted value obtained by the RF model.

Then, the coefficient of determination (*R*^2^) between the predicted value and the true value of all OOB data is the OOB score of the model [54]:

(9) $$R^{2}=1-\frac{MSE_{OOB}}{{\hat{\sigma }}_{y}^{2}}$$

where, ${\hat{\sigma }}_{y}^{2}$ is the variance of OOB predicted value.

The higher OOB score of a model get, the greater performance the model will have [49], so we can determine the optimal dimensionality (number of features) of the model based on the OOB score. 

Different from cross-validation, in the OOB score calculation process, the model is tested on OOB samples, which means that these data was not used in any previous data training; so we can easily avoid data leakage problem and acquire a more accurate prediction model with little over-fitting and minimum variance. However, OOB is a random sampling based on random forest, hence there can be randomness in results acquired from OOB calculation; and the best way to tackle this randomness is conducting multiple trial to gain more stable results [55].

To sum up, according to respective characteristics of RFE and OOB, this study combines OOB with RFE in the random forest to form an OOB RFE method, which excelled both traditional methods in feature optimization. Here is the principle of OOB RFE: Setting RFE as algorithm framework, we firstly extracted several samples through bootstrap re-sampling of random forest to form OOB; then, decision tree was constructed for each bootstrap sample to constitute a random forest, which was applied in recursion model for feature importance calculation; In the meantime, we introduced cycle iteration of RFE to evaluate the relevance of features and eliminate the features with relative low importance; After that, we conducted random forest repeatedly to calculate the remaining features until one feature left, and could easily select the optimal feature combination among all features through comparing their determination coefficient and root-mean-square error. 

**Figure 9:**
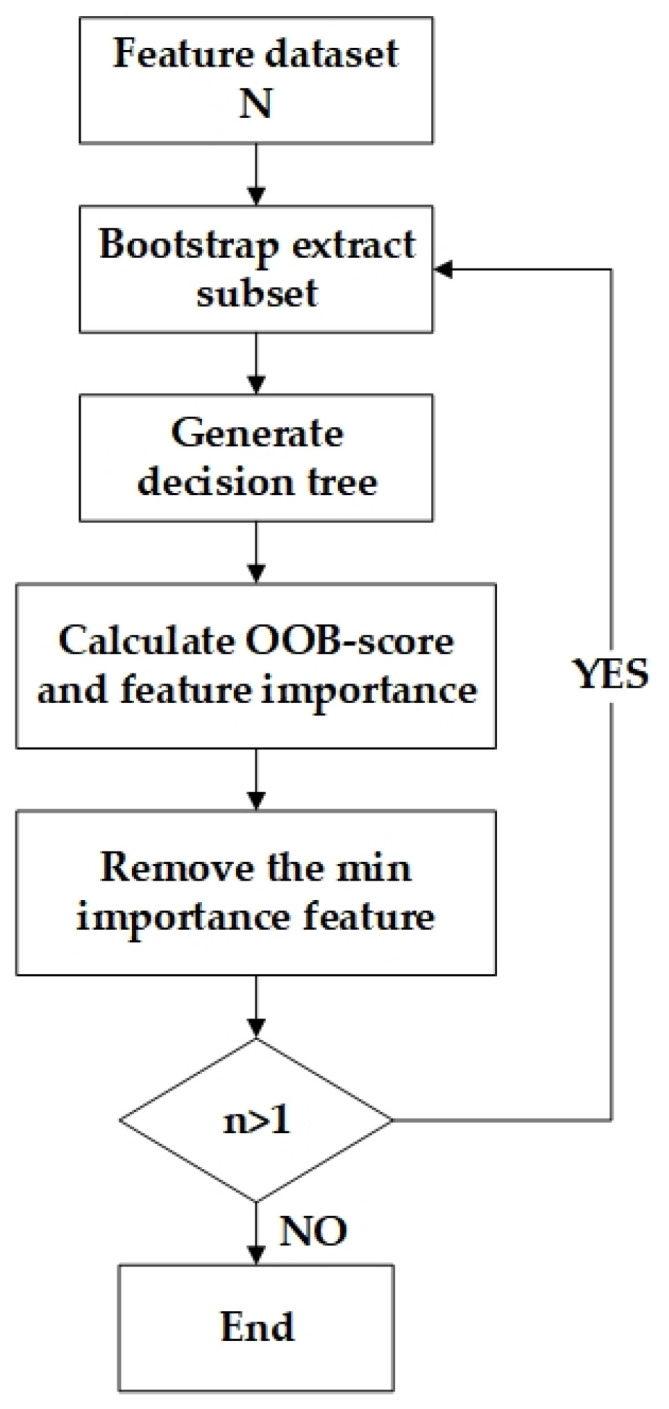
Flow chart of Out of Bag data with Recursive Feature Elimination (OOB RFE).

The detailed process is shown as following (Figure 9):

(1) Starting with an initial feature dataset N with n features, this study constructs a regression tree using subsets extracted by bootstrap random sampling and gathers the OOB data to form a test sample
(2) According to certain criteria, the optimal branch was chosen from regression tree, which allows the maximum growth of each decision tree
(3) After integrating the regression tree in (1) to build a random forest regression model, we can calculate OOB score and obtain feature importance based on OOB error
(4) According to the principle of backward iteration, we can delete the feature with the smallest feature importance
(5) The whole process (1)–(4) has been repeated over and over until one feature left. After data output, we select the number of features that generates the largest OOB score as the optimal feature number, and select variables based on the feature importance ranking to form the optimal feature combination.

It can be seen that our OOB RFE, compared with traditional RFE, differentiates data set by sampling OOB randomly before feature elimination, which directly applies OOB in final result validation without involving OOB in model training; while all data are included in cross validation of RFE. Therefore, by effectively avoiding the problem, that same data repeatedly was used in training model and validation, namely data leakage problem, this new method successfully guarantees the generalization ability of the model. In addition, setting RFE as algorithm framework, our OOB RFE method can reevaluate remaining features in each cycle iteration, so the score of each feature is adjusted during the iteration; which overcomes the shortage of OOB optimal feature selection that repetitious trials are required to gain stable data subset.

### 3.3. Feature Combination Scheme

In order to compare the classification effects obtained by different feature combinations, Table 4 shows six sets of feature combination schemes in this study. Scheme 1 was based on the spectral features and texture features extracted by ZY-1-02D. Scheme 2 added LST, which extracted from Landsat-8 to the Scheme 1. Scheme 3 was the combination of Scheme 1 and the polarization and texture features obtained from Sentinel-1. Scheme 4 contained all the features based on 3 types of data; Scheme 5 was the OOB RFE optimization of Scheme 4′s feature combination. Scheme 6 was an optimized combination of Scheme 4 using traditional RFE, base model of which is SVM, using 2-fold cross-validation to determine the number of features. Scheme 6 was mainly used to compare and evaluate the effect of OOB RFE’s result.

### 3.4. Classification Algorithm and Samples

In recent years, machine learning algorithms have good performance in remote sensing image classification [56,57,58]. SVM and RF are especially two typical and commonly used examples among them [59,60,61]. This study used the 2 classification methods above to classify and extract the mineral exploiting information in the study area by the ENVI software program, and the algorithms’ parameter settings are demonstrated in Table 5.

This study used random sampling based on LULC data and results of field research to extract 1100 samples that could be applied in classification and accuracy assessment (Table 6). We doubled the samples of mines to ensure the accuracy of mining information excluding any other disruption.

### 3.5. Accuracy Assessment

This study adopted a prevailing method ‘confusion matrix’ to verify the accuracy of the classification results. The evaluation factors obtained by the confusion matrix include: overall accuracy (OA), kappa coefficient, producer accuracy (PA), and user accuracy (UA).

## 4. Results

### 4.1. Optimized Results and Feature Importance

This research ran sample data in the Python sklearn module to optimize the feature combination. Through OOB RFE calculation, the model had the highest OOB score 0.9162 when the number of features equaled to 36. Moreover, through cross-validation, the RFE model got peak value 0.9058 when the number of features was 41. Therefore, the optimal number of features obtained by OOB RFE and RFE were 36 and 41, respectively.

**Figure 10:**
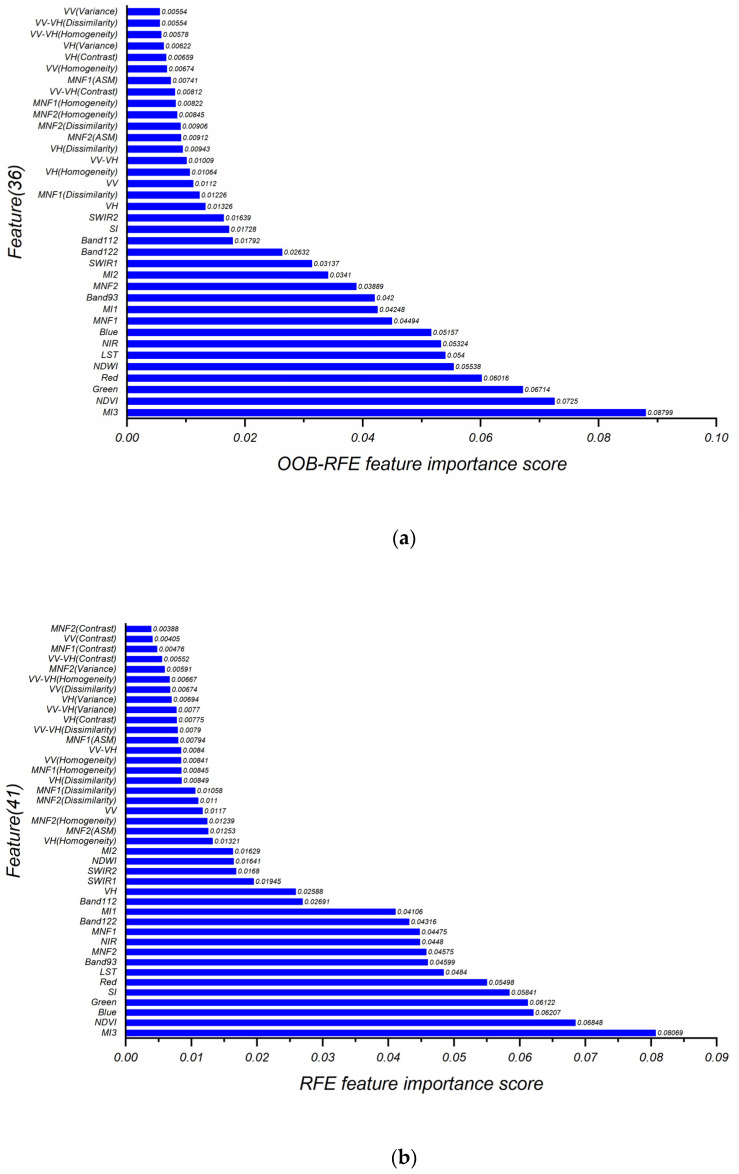
Feature optimization result and importance rank based on: (**a**) OOB RFE, (**b**) RFE.

Figure 10 shows the specific feature variables and their importance scores obtained from OOB RFE and RFE.

It can be seen from Figure 10 that the top 2 importance features among the feature optimization results obtained by the 2 methods were MI3 and NDVI, and the last ranking feature was texture feature. The highest ranked 36 of the feature variables obtained by RFE were generally consistent with which obtained by OOB -RFE, and only slightly different in order of features’ importance. The optimized features from OOB RFE contained VV (Variance) that were excluded in the RFE result, while the RFE result had extra variables VV-VH(Variance), VV(Dissimilarity), MNF2(Variance), MNF1(Contrast), VV(Contrast), MNF2(Contrast) compared with OOB RFE’s.

### 4.2. Accuracy Assessment and Comparison

Applying with SVM, this study used RF algorithms to classify the six feature combination schemes, and got the overall accuracy of classification based on the verification sample (kappa coefficient are shown in Table 7). Scheme 5 optimized by the OOB RFE using RF had the best classification effect with OA of 93.64% and kappa coefficient of 0.926, respectively. In terms of classification method, the OA and kappa coefficient of the RF-based classification outperformed the results of SVM-based classification, except for results in Scheme 1 that overall accuracy difference between this two classification is less than 0.68%. The study presents that the overall accuracy of RF in the rest of the schemes exceeded SVM by more than 1%. In addition, we got the lowest OA of 81.48% and kappa coefficient of 0.785 by using Scheme 1 (only ZY-1-02D dataset) to extract mineral exploiting information; the OA and kappa coefficient of Scheme 2 and Scheme 3 have been greatly improved than results of Scheme 1. It indicated that the addition of Landsat-8 based LST features and Sentinel-1 based polarization and texture features effectively increase the classification accuracy of mineral exploiting information. The average OA obtained from Scheme 4, which included all feature variables, was 90.35% and the kappa coefficient reached 0.888. Furthermore, through feature optimization, the OA of Scheme 5 and Scheme 6 had further increases than OA of Scheme 4. Comparatively, Scheme 5 optimized by OOB RFE had achieved better classification effects than Scheme 6 as the OA improved by 0.68%.

**Figure 11:**
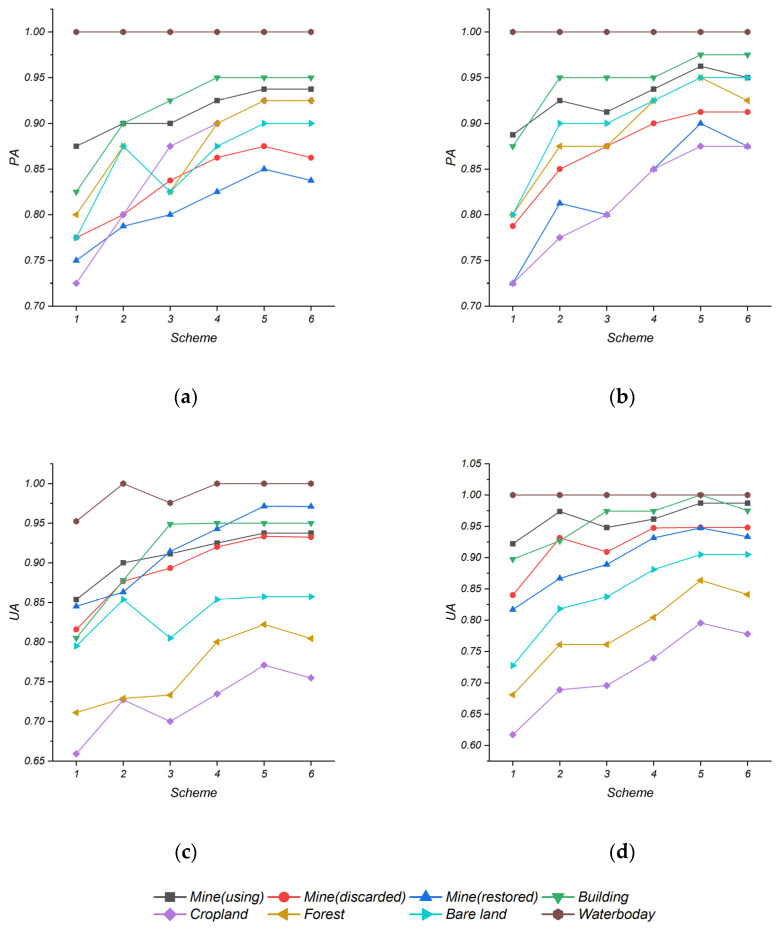
(Top) Producer accuracy (PA) of each type by (**a**) SVM, (**b**) RF; (Bottom) User accuracy (UA) of each type by (**c**) SVM, (**d**) RF.

Figure 11 shows the PA and UA of land features under different scenarios and methods. The water body had a very stable and high PA and UA in each scheme due to its disparate differences from other land features. In general, both the PA and UA of the objects would gradually increase as we tested schemes 1 to 5. The PA and UA of the ground objects obtained by Scheme 1 with a single ZY-1-02D spectral characteristic, especially in the croplands, forests, discarded, and restored mines, were the lowest due to their spectral similarities. After adding LST, polarization, and texture to Scheme 2 and Scheme 3, the PA and UA both increased even with different degrees. It is noteworthy that some objects of this study (like, recovered mines, forests, and croplands) received better accuracy in Scheme 2 than in Scheme 3 because of the characteristic difference of those land features, so we could conclude that their LST feature differences were more distinctive than polarization and texture features. On the contrary, using mines and bare lands with sensitive polarization and texture features would show higher accuracy in Scheme 3. The accuracy further increased in Scheme 4 including all features. Furthermore, Scheme 5 and Scheme 6 both obtained higher accuracy in PA and UA than Scheme 4 after feature optimization. Compared with Scheme 6, Scheme 5 had a better classification effect on the restored mines, cropland, and forest.

**Figure 12:**
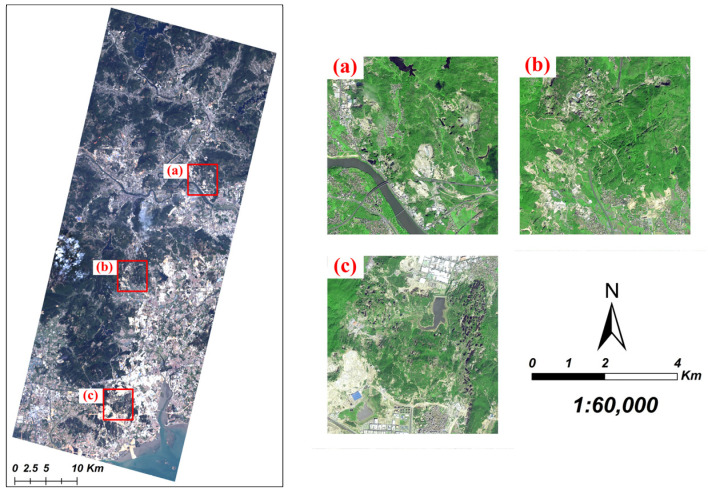
Locations and high resolution images (from Gaofen-2 satellite) of 3 selected areas used for visual verification: (**a**) Northern mineral exploiting area, (**b**) Middle mineral exploiting area, (**c**) Southern mineral exploiting area.

**Figure 13:**
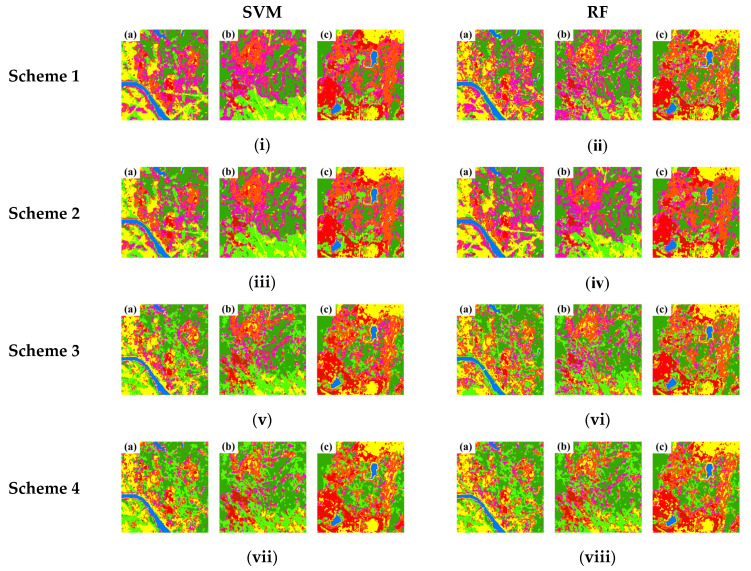

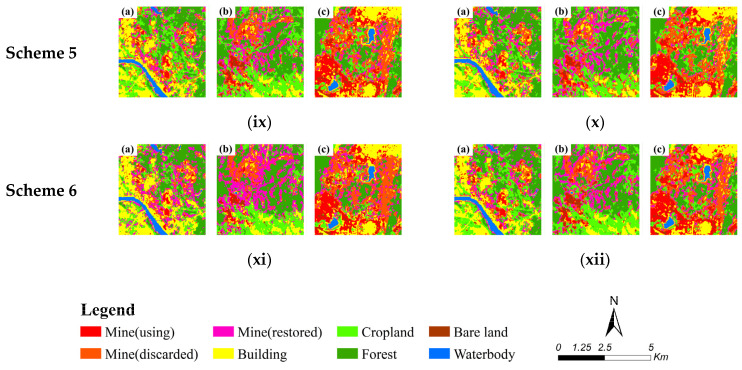
Comparison of classifications from: (**i**) Scheme 1 by SVM, (**ii**) Scheme 1 by RF, (**iii**) Scheme 2 by SVM, (**iv**) Scheme 2 by RF, (**v**) Scheme 3 by SVM, (**vi**) Scheme 3 by RF, (**vii**) Scheme 4 by SVM, (**viii**) Scheme 4 by RF, (**ix**) Scheme 5 by SVM, (**x**) Scheme 5 by RF, (**xi**) Scheme 6 by SVM, (**xii**) Scheme 6 by RF; (**a**), (**b**), (**c**) represent the selected northern, middle and southern mineral exploiting area in the study field, respectively.

In addition, three mine concentration areas in the study area (Figure 12) were used to evaluate the visual effects of the classification results obtained from different methods and schemes, which are shown in Figure 13. It can be seen in all schemes that the features of water body were accurately extracted. However, in Scheme 1, there were high-frequent misclassifications among discarded mines, restored mines, croplands, and forests appearing in result, and some built-up areas and bare land in c were misclassified as using mines. Scheme 2′s result became more accurate in distinguishing restored mines, forests, and discarded mines, but it had a bad performance in identifying using mines, buildings, and bare lands in flatland. The result of Scheme 3 showed high distinctive results to effectively distinguish discarded mines, bare lands, and built-up areas. Moreover, the classification effects of Scheme 4, Scheme 5, and Scheme 6 are close to each other, but Scheme 5 and 6 displayed more details when extracting the land features’ boundaries. Scheme 5 especially showed little speckle noise in image. Comparing those classification methods, it is evident that the RF-based classification that obtained more complete features and divided small features more accurately has the better overall visual effect than SVM.

## 5. Discussion

Due to the structural complexity and high heterogeneity, classifying mineral features through single dataset should be a challenge [62], while we refer complementary multi-sensor satellite images to separate mineral features from non-mineral features. The results of this study are consistent with previous research on mapping classification by integrating multi-source data [63,64], which indicates that combining ZY-1-02D hyperspectral imagery with the heat and polarization characteristics provided by Landsat-8 and Sentinel-1 data can effectively enable us to identify mining information in the study regions and acquire accurate results. Specifically, the combination of hyperspectral features and LST inversed from thermal infrared data (Scheme 2) outperforms the single spectral feature in distinguishing restored mines, forests, discarded mines, and croplands, which have highly similar spectral curves, from thermal perspective; however, when it comes to plains, the combination is not ideally functioned on identifying using mines, built-up areas, and bare lands. The results show that the LST-based classification difference is both physical and logical [28]. As a result, through appropriate inversion combined with other features, LST feature obtained from Landsat-8 OLI+TIRS can be used to improve the classification accuracy. The combination of hyperspectral and SAR features (Scheme 3) that has similar classification accuracy with Scheme 2 can better extract used/discarded mines and bare lands with different backscattering coefficients. The combination of all feature sets (Scheme 4) obtained from the three types of remote sensing images has achieved more accurate classification results than the two data combinations (average overall accuracy increased by 9.38%, the corresponding kappa coefficient is increased by 0.108).

Through OOB RFE and RFE feature combination optimization, Scheme 5 and Scheme 6 not only cut down the number of required features (36 and 41, respectively), but also has higher classification accuracy than Scheme 4. The optimization applied in the classification of mineral exploiting information practically reduces the redundancy of feature information while enhancing the classification efficiency and accuracy. Furthermore, differing from Scheme 6, Scheme 5 further improves the overall accuracy and the classification accuracy of restored mins, croplands, and forests. Besides, compared with the RFE method, the OOB RFE method proposed in this study can generate better results with less optimized feature combinations. It indicates that our method successfully overcomes the shortages of RFE in feature selection and extracts more accurate and effective features.

According to the results of feature importance (Figure 9), there are only seven differences between the two feature selection methods. Among all features, spectral features as basic information makes the largest contribution to land-cover information classification while LST respectively ranks sixth and seventh in the two results. The mines under different developing status have spectral similarities with other landscapes, whereas great thermal differences because of human activities, correspond with the physical truth and conclusions of previous studies [19]. Polarization characteristics of SAR, VH, and VV both rank relatively high, because backscattering coefficients of polarization data can be easily distinguished in research of mines, built-up areas, and bare lands [65]. However, the texture feature has low ranking. The possible reason is that we generally used the medium-resolution images (30 m) that lack delicate details to identify land information by their texture.

In terms of classification methods, under the same feature combination scheme, the overall accuracy of the RF classification exceeds that of SVM. The RF method compared with SVM shows greater stability, namely that RF method is unlikely to overfit as processing multi-dimensional data and multi-type classification. Furthermore, it can display clear boundaries between different land features in mineral exploiting research, which enable us to extract fine details. Even though slight differences existed in classification accuracy obtained by the two mentioned methods using single feature data, in general, for the classification of mineral exploiting information in our study area, the RF algorithm certainly can filter the optimal classification and has strong robustness.

## 6. Conclusions

Based on a total of 52 features including spectrum, heat, polarization, and texture features, respectively, extracted from ZY-1-02D, Landsat-8, and Sentinel-1, this study creatively proposed an OOB RFE feature combination optimization method and integrates it with RF and SVM classification methods to effectively classify mineral exploiting information. After assessing and comparing the accuracy and visual of different feature combination schemes, we draw the conclusion as below:

- In this study, using the traditional RFE algorithm and the OOB RFE method to perform dimensional reduction and feature optimization, we decided the number of features included in optimal combination should be 41 and 36, respectively. It was shown that the average OA of these two classification increase 1.365 and 2.05%, respectively. Also, it was noteworthy that the classification based on OOB RFE method had top performance, as it gained the highest average OA 92.39%. Comparing with traditional RFE method, OOB RFE method could reduce more data redundancy and enhance model generalization ability, so it could make a balance between feature dimension and classification accuracy of mineral exploiting information. In addition, compared with SVM, RF, average OA, of which rises 1.78%, could more accurately distinguish the land boundary of mineral exploiting information and get stronger robustness.
- Among the importance gained by two feature optimization, specifically for the mineral exploiting information in our study, spectral features based on ZY-1-02D showed the highest importance, while features like MI3 and NDVI rank in the second place; and LST gained from Landsat-8 successively ranked in sixth and seventh in two different methods; besides, the importance of other polarization features, like VV, VH from Sentinel-1 got a middle position in our ranking; However, the texture feature had the lowest importance as it ranks 20th among all features.

To conclude, our study can provide a usable reference about the selection of optimal features for classification of open-pit mineral exploiting information in other areas or other mines.

## Figures and Tables

**Table 1:** Characteristics of satellite images.

Data	ZY-1-02D	Landsat-8	Sentinel-1
Spatial resolution (m)	30	Operational land imager: 30 (Panchromatic band: 15) Thermal infrared Sensor: 100	Ground Range Detected (GRD): 20 × 22
Bands information	Visible~near infrared (0.40–1.04 µm): 76 bands Short wave infrared (1.00–2.50 µm): 90 bands	Visible~Short wave infrared (0.40–2.29 µm): 9 bands (include 1 panchromatic band) Thermal infrared (10.6–12.51 µm): 2 bands	Dual VV + VH polarization *
Acquisition date	16 March 2020	16 March 2020	17 March 2020
Cloud cover	3%	1.3%	-

* VV: vertical transmit and vertical receive; VH: vertical transmit and horizontal receive.

**Table 2:** Band features of ZY-1-02D reference to Landsat-8 Operational Land Imager (OLI).

Band Feature	Related Bands’ Wavelength of ZY-1-02D (nm)	Landsat-8 OLI Wavelength Width (µm)
Blue	455, 464, 473, 481, 490, 499, 507	0.45–0.51
Green	533, 542, 550, 559, 567, 576, 585	0.53–0.59
Red	645, 653, 662, 670	0.64–0.67
NIR	851, 859, 868, 876	0.85–0.88
SWIR1	1576, 1593, 1610, 1627, 1644	1.57–1.65
SWIR2	2115, 2132, 2148, 2165, 2182, 2199, 2216, 2233, 2249, 2266, 2283	2.11–2.29

**Table 3:** Spectral Index Features used for Classification.

Index Name	Calculation Formula
Enhanced Vegetation Index (EVI)	$2.5\left(\frac{\rho_{\mathrm{NIR}}-\rho_{\mathrm{red}}}{\rho_{\mathrm{NIR}}+6\rho_{\mathrm{red}}-7.5\rho_{\mathrm{blue}}+1}\right)$
Soil Index (SI)	$\frac{\left[\left(\rho_{\mathrm{SWIR}1}+\rho_{\mathrm{red}}\right)-(\rho_{\mathrm{NIR}}+\rho_{\mathrm{blue}}\right)]}{\left[\left(\rho_{\mathrm{SWIR}1}+\rho_{\mathrm{red}}\right)+(\rho_{\mathrm{NIR}}+\rho_{\mathrm{blue}}\right)]}$
Normalized Difference Water Index (NDWI)	$\frac{\left(\rho_{\mathrm{green}}-\rho_{\mathrm{NIR}}\right)}{\left(\rho_{\mathrm{green}}+\rho_{\mathrm{NIR}}\right)}$

Where, $\rho_{\mathrm{blue}}$, $\rho_{\mathrm{green}}$, $\rho_{\mathrm{red}}$, $\rho_{\mathrm{NIR}}$, $\rho_{\mathrm{SWIR}1}$ respectively represent the blue, green, red, near-infrared, and short-wave infrared 1 band values.

**Table 4:** Feature combination schemes used for classification.

Scheme	Layers
1	ZY-1-02D: 2 MNF, 15 band-index features, 10 textures.
2	Scheme 1, Landsat-8: 1 LST.
3	Scheme 1, Sentinel-1: 4 Polarimetric features, 20 textures.
4	Intersection of Scheme 2 and Scheme 3.
5	OOB RFE combination based on Scheme 4.
6	RFE combination based on Scheme 4.

**Table 5:** Parameter settings of 2 classification algorithms.

**Support Vector Machine (SVM)**	**Kernel Type**		**Gamma**		**Penalty Parameter**		**Pyramid Levels**	
	Radial basis function		1/Nvar *		100		0	
**Random Forest (RF)**	**Number of Trees**	**Number of Features**		**Impurity function**		**Min Node Samples**		**Min Impurity**
	100	Square Root		Gini coefficient		1		0

* 1/Nvar means the inverse of the number of input variables.

**Table 6:** Quantities of samples.

NO.	Types		Quantities of Samples	
			Training	Test
1	Mines	Using	120	80
2		Discarded	120	80
3		Restored	120	80
4	Buildings		60	40
5	Croplands		60	40
6	Forests		60	40
7	Bare lands		60	40
8	Water bodies		60	40

**Table 7:** Overall accuracy (OA) and kappa coefficient of each classifications.

	SVM		RF	
	OA	Kappa	OA	Kappa
Scheme 1	81.14%	0.781	81.82%	0.789
Scheme 2	85.68%	0.834	87.95%	0.860
Scheme 3	86.59%	0.844	88.18%	0.863
Scheme 4	89.55%	0.878	91.14%	0.897
Scheme 5	91.14%	0.897	93.64%	0.926
Scheme 6	90.68%	0.892	92.73%	0.915
